# Intraoperative pediatrics hypothermia and its factors during general anesthesia at comprehensive specialized hospitals, Northwest Ethiopia: Multicenter follow up study

**DOI:** 10.1371/journal.pone.0320950

**Published:** 2025-05-06

**Authors:** Melese Tadele Mekonnen, Salh Yalew Mustofa, Yophtahe Weldegerima Berhe, Nurhusen Riskey Arefayne, Biresaw Ayen Tegegne

**Affiliations:** 1 Department of Anesthesia, Boru Meda hospital, Wollo, Ethiopia; 2 Department of Anesthesia, School of medicine, College of Medicine and Health Sciences, University of Gondar, Gondar, Ethiopia; Stanford University School of Medicine, UNITED STATES OF AMERICA

## Abstract

**Background:**

The human body can regulate its internal temperature under physiological conditions by balancing heat production and loss. Many pediatric patients may experience hypothermia during surgery and anesthesia. Hypothermia is defined as a core body temperature below 36 °C in pediatric patients undergoing surgery. Therefore, this study was aimed to determine the incidence of intraoperative pediatric hypothermia and its factors during general anesthesia at Comprehensive Specialized Hospitals, Northwest Ethiopia.

**Methods:**

A multi-center prospective follow-up study was conducted from May 2 to July 28, 2023, on 403 participants. A consecutive sampling method was used to select the study participants. Preoperative temperature and initial intraoperative temperature were measured using tympanic thermometer. Descriptive data was carried out and the results were presented as table, text and graph. Data was entered to Epi-data software (version 4.6) and analyzed with STATA software (version 17 SE). Bivariable and multivariable logistic regression analyses were used to identify associated factors. A p-value less than 0.05 with a 95% confidence interval were considered statistically significant.

**Result:**

The incidence of intraoperative pediatric hypothermia was 44.7% (CI=39.8–49.6). The neonates (AOR=3.7,95%CI=1.5–10.8), use of intravenous fluid>500ml (AOR=2.3,95%=1.48–4.43), having blood transfusion (AOR=2.7,95% CI=1.30–5.91), operation room temperature ≤21 degree Celsius (AOR=9.3, 95% CI= 5.78–20.58), operation room temperature in between 22–24^0^c (AOR=2.6, 95%CI=1.28–5.35) and preoperative core temperature≤ 35.9 degree Celsius (AOR=4.8,95%CI=2.42–9.68) were associated with intraoperative pediatric hypothermia during general anesthesia.

**Conclusion and recommendation:**

This study showed that the incidence of intraoperative hypothermia among pediatric surgical patients was considerably high. Neonate, using more than 500ml of intravenous fluid, having blood transfusion, preoperative core temprature≤35.9 °C and operation room temperature ≤21 °C were associated with intraoperative hypothermia during general anesthesia.

Pediatric patients should be monitored their core body temperature during the intraoperative period to prevent and treat hypothermia. Emphasis should be given for neonates. Warming the operating room is recommended. We also recommend to use the optimal fluid and blood transfusion.

## Introduction

Hypothermia is defined as a core body temperature of <36 °C for pediatric patients undergoing surgery and most of the patients may experience hypothermia during surgery under general anesthesia [1,2]. Core body temperature which is measured by tympanic ear thermometer is relatively reliable and accurate than others [3]. The human body regulates its internal temperature under physiological conditions by balancing heat production and loss, but this compensatory regulation can be impaired during surgery [4,5)]. Hypothermia can be categorized into three: mild (35–35.9 °C), moderate (34–34.9 °C) and severe (33 °C) [6,7]. Pediatric patients who undergo surgery may experience hypothermia because of impaired thermoregulation, greater heat loss to the environment, cooling effect of cold anesthetic gases, and decreased body heat production [8].

Heat can be loss during surgery by radiation, convection, conduction, and evaporation [8,9]. The incidence of intraoperative hypothermia during pediatric surgery is 70% [10]. Intraoperative hypothermia might be more pronounced in neonates, infants, toddlers, and school-age children due to their high surface area-to-weight ratio, greater susceptibility to heat loss, lack of subcutaneous fat storage, and reduced thermoregulatory capacity [11]. The high incidence of intraoperative hypothermia was associated with age, type of anesthesia, types of surgery, duration of surgery, systemic disease, amount of fluid/blood use, temperature of operation room and patients’ preoperative hypothermia [12–15].

Untreated hypothermia can result prolonging the action of anesthetic medications, impairing coagulation, increasing blood loss, increasing the risk of wound infections, hypoglycemia, hypoxia, prolonging hospital stays, causing postoperative discomfort, shivering, increasing heart rate, blood pressure, and plasma catecholamine levels [12,16,17].

Even though the use of passive warming is used throughout surgery, the occurrence of intraoperative hypothermia in pediatrics is increasing [17]. The preprocedural warming of the patient in addition to intraoperative passive warming minimized intraoperative hypothermia [18]. Intraoperative hypothermia can be prevented by warming IV fluids and using methods such as forced-air warming or convection warming to transfer heat to the patient [19,20].

In majority of the studies, intraoperative pediatric patients’ body temperature was measured with axillary thermometer and limited studies have conducted by using tympanic ear thermometer [21]. Therefore, the aim of this study was to determine the incidence of intraoperative pediatric hypothermia and its factors during general anesthesia.

## Method

### Study design and period

A multicentre prospective follow up study was conducted at comprehensive specialized hospitals, Northwest Ethiopia, May 02 to July 28,2023.

### Study area

The study was conducted at four comprehensive specialized hospitals in Northwest Ethiopia, which are the University of Gondar, Tibebe-Ghion, Felege-Hiwot, and Debre Markos. UOG is located in central Gondar administrative zone, Amhara national regional state which is far from Addis Ababa by about 740 km. University of Gondar comprehensive specialized hospital has the main operation rooms, obstetrics operation rooms, ophthalmologic operation rooms and gynecology operation rooms. Felege Hiwot and Tibebe Gion hospitals are found in Bahir Dar by about 565 km far from Addis Ababa. Both TGCSH and FHCSH have nine and six major operation rooms respectively. Debre Markos Hospital has found in Debre Markos city which is 296km far from Addis Ababa. Debre Markos Hospital has four operation rooms. The number of pediatric surgical patients are increasing in above hospitals.

### Source and study population

#### Source population.

All surgical pediatrics patients who took anesthesia under surgery.

#### Study population.

All surgical pediatric patients who took general anesthesia till the calculated sample size were reached.

### Eligibility criteria

#### Inclusive criteria.

All surgical pediatrics patients undergoing surgery by general anesthesia.

#### Exclusion criteria.

Patients who had fever, general anesthesia lasting less than 30 minutes, bilateral meatus obstruction that can be ruled out with mastoid and tragus tenderness, open cardiothoracic surgery and pediatric patients whose parents were not present.

### Variables

#### Dependent variable.

Intraoperative pediatrics hypothermia

#### Independent variables.

Sociodemographic and clinical characteristics: age, sex, ASA status, weight, preoperative core temperature, and coexisting disease.

Intraoperative related factors: Types of anesthesia, duration of anesthesia, medication, use of a caudal block, types of procedures, the urgency of surgery, duration of surgery, operation room temperature, volume of iv fluid used >500 ml, blood transfusion, warmed fluid administered

### Operational definition

Normothermia is defined as a core temperature between 36 °C and 38 °C [22,23].

Intraoperative hypothermia is defined as a core temperature less than 36 degree Celsius during the time intervals [22,23].

Hyperthermia is defined as a core temperature of more than 38 °C [22,23].

Pediatrics is defined as an age ≤12 years [22].

Operation room temperature is measuring the temperature of a specific operating room

Ambient temperature is measuring the temperature of the entire operating theater.

### Sample size and sampling technique

#### Sample size.

Single population proportion formula was used to calculate the sample size by considering 95% CI, and a 5% margin of error. The proportion of intraoperative hypothermia (39.9%) was used from the previous study done in Tikur Anbesa specialized hospital [24]. By using a single population proportion formula; n= (Z a/2)2×p×q/d2


$$\frac{\text{(}\left(\text{1.96}\right)\text{2X}\left(\text{0.4}\right)\left(\text{0.6}\right)}{\text{(0.05)2}}\text{=369}$$

Where: n= sample size.

Z= desired 95% confidence, Z=1.96.

*p* = proportion of hypothermia (0.4)

*q* =1-*p = 1-0.4=0.6*

*d =* is the margin of sampling error tolerated (5%)

By considering 10% non-response rate the total sample size is 406.

#### Sampling technique.

**Fig 1:**
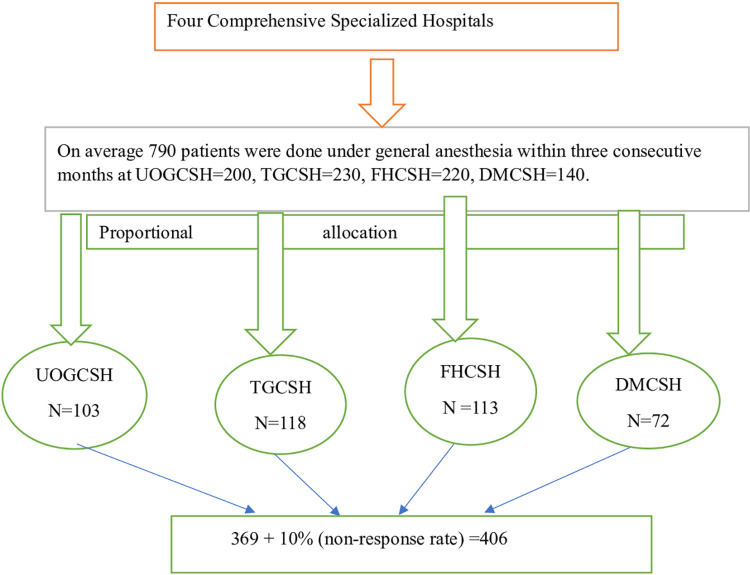
Flow diagram of study participants in comprehensive specialized hospitals of Northwest Ethiopia.

All consecutive pediatric surgical patients who underwent surgery were obtained from UGCSH, TGCSH, DMCSH, and FHCSH operation theatres during the intraoperative period. The prospective follow up study was conducted based on single proportional allocation. A total of 790 paediatric surgical patients were done in the last three consecutive months (Fig 1).

### Data collection tool and procedure

A semi-structured questionnaire was used as a data collection tool based on the review of the literature [22,25,26]. Responsible anesthetist was trained by principal investigator and then the data were measured and refilled the questions. The questions were written in English version. Temperature was measured by infrared tympanic thermometer before induction and immediately after 30-minute induction of anesthesia, then every 30 minutes until the procedure finished as representative of core temperature. Room temperature of the operation room was recorded through a room thermometer after all basic monitoring were applied.

### Data quality control

Data was collected by anesthetists guided by 4 supervisors. The training was given to data collectors. A pretest was conducted at the University of Gondar Comprehensive Specialized Hospital among 20 participants, who were not included in the main study. The collected data was checked for completeness, accuracy, and clarity by the principal investigator. The Supervisor as well as the principal investigator supervised the data collectors and checked for the completeness of the data daily.

### Data processing and analyzing

After completion of data collection, the variables were entered, coded, and cleaned for errors using Epi-data software (version 4.6). Then the data was transformed into STATA software (version 17 SE) and analyzed using STATA software (version 17 SE). Descriptive data were carried out and the results were presented as table, text and graph. Median and interquartile range were used for variables such as age, weight and temperature.

Both binary and multiple logistic regressions were used to identify factors associated with intraoperative hypothermia of patients undergoing surgery. The p-value <0.2 in binary logistics regression with 95% CI was analyzed with multivariable logistic regression. In multivariable logistic regression analysis, a p-value less than 0.05 with a 95% confidence interval were considered statistically significant. Multi-collinearity and model goodness of fit were checked by the variance inflation factors (VIF) and Hosmer Lem show test respectively.

The data distribution normality was checked by the Shapiro-Wilk test. Chi-square test determined the association between independent factors and the outcome variable at a 95% confidence interval. The Adjusted odds ratio (AOR) with the corresponding 95% confidence interval used to determine the strength of associated independent factors with the outcome variable.

### Ethical consideration

Ethical clearance and approval were obtained from the Ethical Review Committee of the School of Medicine, College of Medicine and Health Science, University of Gondar. Permission to conduct the study was obtained from four Comprehensive specialized referral hospitals. Informed oral consent was obtained from their parents. If the patient did not have an attendant, consent was taken as per anesthesia and surgical team. The data obtained were used only for the study purposes. Confidentiality and anonymity were ensured.

## Result

### Sociodemographic and clinical characteristics of the study

A total of 403 participants were enrolled with a response rate of 100%. The median age of the participants was 4 years with an interquartile range of (IQR= 1,7) years. Of the participants, about half (50.3%) were males. Among the total participants, around 2/3^ed^ (65.51%) have done by elective surgeries. From the total of 403 study participants, about 81(20.1%) of their preoperative body temperature were less than 36 degrees Celsius (Table 1).

**Table1:** Sociodemographic and clinical characteristics of study participants at comprehensive specialized hospitals, Northwest Ethiopia, May 02 to July28/2023 (n=403).

Variable	Category	Frequency	Percentage (%)
Age	Neonate	36	8.9
	Infant	75	18.6
	Toddler	152	37.7
	School-age	140	34.7
Sex	Male	203	50.4
	Female	200	49.6
Weight	<11 kg	153	37.9
	11–20 kg	151	37.5
	21–30 kg	85	21.1
	>30 kg	14	3.5
ASA	ASA I	231	57
	ASA II	162	40
	>ASA III	10	2.5
Coexisting disease	Yes	66	16.4
	No	337	83.6
Preoperative core body temperature	≤ 35.9 c^0^	81	20.1
	≥ 36.0 c^0^	322	79.9

ASA: American society of anaesthesiology

### Intraoperative surgical and anesthesia-related factors of hypothermia

The majority 343 (85.1%) of the study participants underwent surgery under general anesthesia with ETT. Around 156 (38.7%) of the study participants were induced with ketamine. The duration of surgery and anesthesia for more than half of the study participants were less than 70 minutes and less than 90 minutes respectively (Table 2).

**Table 2:** Intraoperative surgical and anesthesia related factors associated with hypothermia during general anesthesia among pediatric surgical patients in comprehensive and specialized, Northwest Ethiopia from May02 to July 28, 2023.

Variable	Category	Frequency	Percentage (%)
Urgency of surgery	Emergency	149	34.5
	Elective	254	65.5
Type of procedure	Orthopedic	65	16.6
	Abdominal surgery	139	34.5
	Urologic surgery	56	13.9
	Ear, neck, and throat	70	17.3
	Ophthalmic	24	5.96
	Neurosurgery	43	10.7
	Other procedure	7	1.5
Induction agent	Ketamine	156	38.7
	Propofol	131	32.5
	Ketamine and propofol	116	28.8
Type of anesthesia	GA with ETT	343	85.1
	GA with FM	28	9.4
	GA with LMA	22	5.5
Caudal block	Yes	67	16.6
	No	336	83.4
Blood transfusion	Yes	50	12.4
	No	353	87.6
Use of iv fluid >500 ml	Yes	77	19.1
	No	326	80.9
Warm fluid administers	Yes	43	10.67
	No	360	89.3
Inhalational agent	Halothane	145	35.9
	Isoflurane	251	62.3
	Not used	7	1.7
Muscle relaxant	Suxamethonium	177	43.9
	Vecuronium	3	0.7
	Suxamethonium and vecuronium	170	42.2
	Not used	53	13.2
Type of analgesia	Morphine	26	6.5
	Pethidine	112	34.3
	Fentanyl	207	51.5
	Other	57	14.2
Operation room temperature	<22^0^ C	138	34.2
	22–24 °C	73	18.1
	>24 °C	192	47.6
Duration of surgery	<70 min	209	51.9
	>70 min	194	48.1
Duration of anesthesia (min)	<90 min	228	56.6
	>90 min	175	43.4

GA: general anesthesia, ETT: endotracheal intubation, LMA: laryngeal mask airway

### Incidence of intraoperative hypothermia

**Fig 2:**
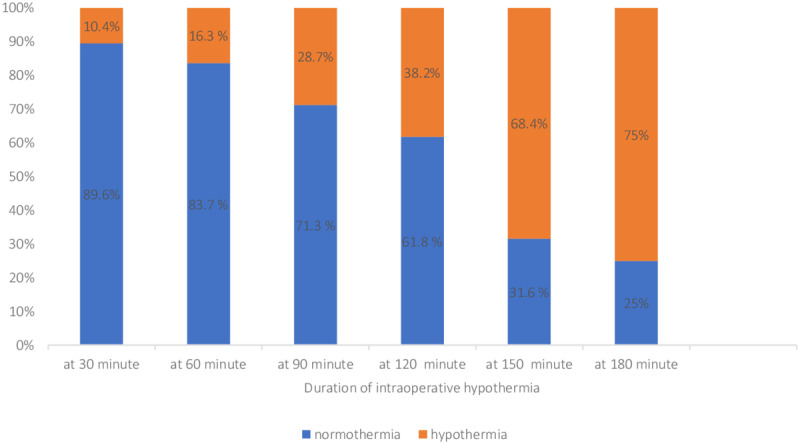
The trend of core body temperature under general anesthesia.

The incidence of intraoperative hypothermia was about 44.7% (CI=39.8–49.6). Around 10.4% of their core body temperature at 30 minutes after induction was less than 36 degrees Celsius. The trend of intraoperative core body temperature under anesthesia was measured at different time intervals and patients became hypothermic after 30 minutes of induction (Fig 2).

#### The median core temperature.

**Fig 3:**
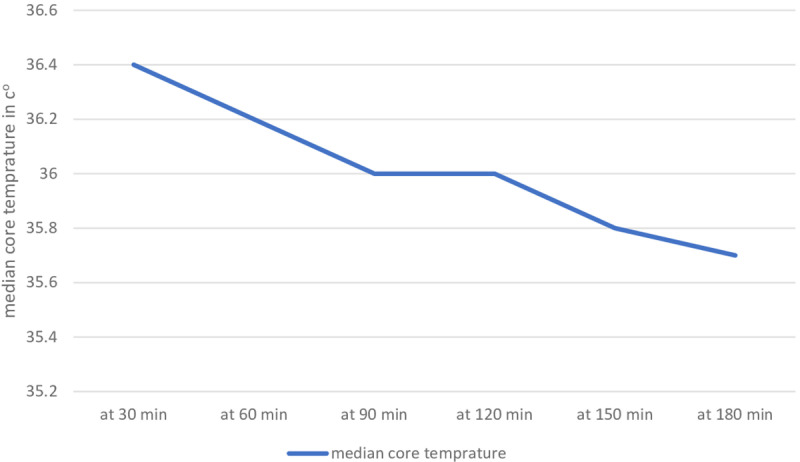
Median core body temperature during general anesthesia at different time interval.

The median preoperative core temperature was 36.5 with an interquartile range (IQR= 36.0–36.7 °C). Median intraoperative body core temperature under general anesthesia at each time interval were at 30 minute 36.4 °C (IQR =36.1–36.6 °C), at 60-minute 36.2 °C (IQR=36–36.4 °C), at 90 minute 36 °C (IQR=35.8–36.2 °C), at 120-minute 36 °C (IQR=35.5–36.4 °C), at 150 minutes 35.8 °C (IQR=35.5–36 °C) and at 180 minutes 35.7 °C (IQR=35.5–35.9 °C) (Fig 3).

### Factors associated with intraoperative hypothermia

In bivariable logistic regression analysis age, the urgency of surgery, type of procedure, caudal block, blood transfusion, duration of surgery, duration of anesthesia, use of more than half ml of IV fluids, preoperative temperature and operation room temperature were associated with intraoperative hypothermia with p-value of <0.2. Neonate, use of more than half ml of IV fluid, having blood transfusion, preoperative temperature≤35.9 degree Celsius and operation room temperature ≤21 degree Celsius were associated in multivariable logistics regression at p-value <0.05.

The odd of being hypothermia for neonate is 3.7 times (AOR=3.7, 95% CI = 1.37–10.23) higher than school age groups. The odd of being hypothermia in patients who were received blood transfusion is 2.7 (AOR=2.7, 95% CI=1.24–5.68) times greater than the counter parts. The odd of being hypothermia in patients who were used iv fluid greater than 500 ml is 2.3 (AOR=2.3,95% CI=1.14–4.67) times higher than patients who were used iv fluid less than 500 ml. The odd of being hypothermia in patients who were in operation room temperature ≤ 21 degrees Celsius is 9.3(AOR=9.3, 95%CI=4.82–18.04) times higher than patients who were in operation room temperature of >24-degree Celsius. Pediatric patients with the operation room temperature in between 22–24 °C is 2.6 times (AOR=2.6, 95%CI=1.28–5.35) more likely to develop intraoperative hypothermia than patients who were in operation room temperature of >24-degree Celsius. The odd of being hypothermia in patients who have preoperative temperature of ≤35.9 degree Celsius is 4.8 (AOR=4.8,95%CI=2.42–9.68) times higher than in patients who have no preoperative temperature of >36 °C (Table 3).

**Table 3:** Bivariable and multivariable logistic regression result for factors associated with intraoperative hypothermia during general anesthesia among pediatric surgical patients at comprehensive specialized hospitals, Northwest Ethiopia, May 02 to July 28, 2023 (n=403).

Variable	Category	Intraoperative hypothermia		COR (in 95%CI)	AOR (in 95% CI)	P-value
		Normothermia n (%)	Hypothermia n (%)			
Age	Neonate	11(30.6)	25(69.4)	3(1.4–6.6)	3.7(1.37–10.23)	0.01*
	Infant	48(64)	27(36)	0.75(0.4–1.3)	0.85(0.39–1.83)	0.67
	Toddler School-age	84(55.3) 80(57.1)	68(44.7) 60 (42.9)	1(0.7–1.7) 1	1.1(0.6–2.1) 1	0.79
Preoperative temperature	≤ 35.9 °C	21(25.9)	60(74.1)	4.8(2.78–8.30)	4.8(2.42–9.68)	0.000**
	≥ 36 °C	202(62.7)	120(37.3)	1	1	
Urgency of surgery	Emergency	61(41)	88(59)	2.5(1.67–3.84)	1.6(0.93–2.87)	0.09
	Elective	162(63.8)	92(66.2)	1	1	
Type of procedure	Orthopedic	33(50.8)	32(49.2)	4.8(1.49–15.75)	3.7(0.76–17.788)	0.10
	Abdominal	54(38.8)	85(61.2)	7.9(2.55–24.27)	4(0.89–18.03)	0.07
	Urologic	30(53.6)	26(46.4)	4.3(1.35–14.3)	2.7(0.57–12.83)	0.21
	ENT	54(77.1)	16(22.9)	1.5(0.44–4.9)	2(0.43–9.6)	0.37
	Ophthalmic	20(83.3)	4(16.7)	1	1	
	Neuro	27(62.8)	16(37.2)	2.9(0.85–10.22)	0.68(0.48–2.01)	0.48
	Other	5(83.3)	1(16.7)	1(0.9–11.02)	0.25(0.02–3.01)	0.27
Caudal block	Yes	25(37.3)	42(62.7)	2.4(1.40–4.13)	1(0.45–1.99)	0.91
	No	198(58.9)	138(41.1)	1	1	
Blood transfusion	Yes	15(30)	35(70)	3.35(1.76–6.35)	2.7(1.24–5.86)	0.012*
	No	208(58.9)	145(41.1)	1	1	
Use of iv fluid >500ml	Yes	32(41.6)	45(58.4)	1.98(1.20–3.29)	2.3(1.14–4.67)	0.02*
	No	191(58.6)	135(41.4)	1	1	
Duration of surgery (min)	<70 min	136(65.1)	73(34.9)	1	1	
	>70 min	87(44.8)	107(55.2)	2.29(1.53–3.42)	0.5(0.17–1.62)	0.27
Duration anesthesia(min)	<90 min	147(64.4)	81(35.5)	1	1	
	>90 min	76(43.4)	99(56.5)	2.36(1.57–3.53)	2.8(0.9–8.57)	0.06
Operation room temperature	≤21	30(21.7)	108(78.3)	13.6(8–23.3)	9.3(4.82–18.04)	0.000**
	22–24	41(56.2)	32(43.8)	2.9(1.6–5.1)	2.6(1.28–5.35)	0.008*
	>24	152(79.2)	40(20.8)	1	1	

COR= crude odd ratio, AOR= adjusted odd ratio, CI=95% confidence interval. The “** or *” p-value was statistically highly significant and significant, respectively. 1= reference, IV = intravenous fluid

## Discussion

The incidence of intraoperative pediatric hypothermia under general anesthesia is 44.7%. The finding of the current study is higher than other study [22]. The incidence of intraoperative hypothermia in pediatric patients who underwent surgery was 20.58% in a study done at Turky [(27)]. This is also supported by another study done in Netherlands 28% [28]. The discrepancy might be due to limited use of devise such as fluid warmer and forced air warmer to prevent intraoperative hypothermia in the study setting [29]. The disparity could also be due to seasonal variation, difference in study population, and sample size difference.

A study was conducted in Rwanda with high incidence of intraoperative pediatric hypothermia about 71.7% as compared to our study [26]. The incidence of intraoperative pediatric hypothermia was about 91.6% which is higher than ours [25]. The reason might be due to the utilization of prevention methods like covering of the body with cotton in the current study.

The findings of the current study is comparable to that of another study, which reported an incidence of intraoperative hypothermia among pediatric surgical patients of about 39.9% [24]. This is supported by another prospective survey study, which found an incidence of around 39.9% for intraoperative hypothermia [12]. The similarity in findings might be due to similarities in clinical setup, geographical location, season, and study population. It could also be due to the similarity in intervention protocols for preventing intraoperative hypothermia [30].

In our study, neonates were associated with intraoperative pediatric hypothermia. This study is comparable to a study conducted in Malaysia [17] and Tikur Anbessa [24]. The reason might be explained due to increased heat loss from larger head size, thin skin, lack of subcutaneous fat, and limited ability of compensatory thermogenesis from brown fat.

In this study, the operation room temperature less than 21 degree Celsius and between 22–44 degree Celsius were significantly associated with intraoperative hypothermia. The finding of other studies done in Zambia [25], and Ethiopia [24] was consistent with our study. This is also supported by another study done in India [31]. However, the current findings are contrary to those of a study conducted in Singapore [32]. The disparity might be due to absence of adjusted room temperature to minimize the core body temperature in this study set up.

The findings of the current study indicated that using more than 500 ml of intravenous fluid was significantly associated with intraoperative hypothermia. This is in line with a study conducted in Kenya [22]. This might be due to severe heat loss from cold fluids. In our study transfusions of blood during the intraoperative period were associated with intraoperative hypothermia. This is consistent to a study done in California [11]. This is also supported by a study done in the USA [(51)]. This might be because the duration of surgery increases blood loss and the risk of cold blood transfusion, which may contribute to intraoperative hypothermia. In this study preoperative core body temprature≤35.9 degree Celsius was associated with intraoperative hypothermia. This finding is consistent with study done in China [33] and Egypt [34]. This is also supported with evidence based guideline [28].

## Limitation of the study

We did not able to measure the ambient operation theater temperature.

## Conclusion and recommendation

The incidence of intraoperative hypothermia was considerably high. Neonates, use of more than 500ml of intravenous fluid, having blood transfusion, preoperative core temprature≤35.9 degree Celsius, operation room temperature ≤21 degree Celsius and operation room temperature in between 22–24^0^c were significantly associated with intraoperative hypothermia during general anesthesia.

Pediatric patients should be monitored their core body temperature during the intraoperative period to prevent and treat hypothermia. Emphasis should be given for neonates. Warming the operation room is recommended. We also recommend to use the optimal fluid and blood transfusion.

## Supporting information

S1 Appendix The data which was used for the analysis of this study. (DTA)

S2 Appendix Questioners that incorporates the socio-demographic, clinical characteristics, intraoperative body temperature, surgical and anesthesia factors related to intraoperative pediatrics hypothermia. (DOCX)

S3 Appendix 3 STROBE checklist for cross-sectional studies. (DOCX)

## Abbreviations

AOR: Adjusted odd ratio
ASA: American society of anesthesiology
ASPAN: America Society of peri anesthesia nurse
CI: Confidence interval
COR: Crude odd ratio
DMCSH: Debre Markos compressive specialized hospital
ECC: Ethical clearance committee
ETT: Endotracheal tube
FHCSH: Feleghiwot compressive specialized hospital
OR: Operation room
PA: Pulmonary artery
TGCSH: Tibebeb Gihon compressive specialized hospital
UOGCASH: University of Gondar and specialized hospital
VIF: Variance inflation factors.
