# Supplementary material for: Intraoperative pediatrics hypothermia and its factors during general anesthesia at comprehensive specialized hospitals, Northwest Ethiopia: Multicenter follow up study
Source: PLoS One. 2025 May 6;20(5):e0320950. doi: 10.1371/journal.pone.0320950 (PMC12054851; doi:10.1371/journal.pone.0320950)
Supplement: S2 Appendix — (DOCX) [file pone.0320950.s002.docx]

**Questioners**

A**nnex**

Table 1: Socio-demographic and clinical characteristics of pediatric patients

| No | Variable | category | Go |
| --- | --- | --- | --- |
| 100 | Age of the study participant? |  |  |
| 101 | Sex | 1. Male 2. Female |  |
| 102 | ASA status | ASA1 |  |
|  |  | ASA 11 |  |
|  |  | >ASA 11 |  |
| 103 | Preoperative core temperature | ____________________0c |  |
| 104 | Weight | ______________________kg |  |
| 105 | Coexisting disease | Yes |  |
|  |  | No |  |

Table 2: Intraoperative body temperature of the patients

| 106 | Intraoperative body temperature | At 30 minutes of induction ____0C  At 60 minutes of procedure ____0C  At 90 minutes of surgery _____0C  At120 minutes of surgery______0C  At 150 minutes of surgery_______0C  At 180 minutes of surgery __________0C  Continue every 30 minutes till the surgery complete |  |
| --- | --- | --- | --- |
| 108 | Operation room temperature | ________________________0c |  |

Table 3: Surgical and anesthesia factors related to intraoperative pediatrics hypothermia.

| No | Variables | Category | *Got* |
| --- | --- | --- | --- |
| 201 | Urgency of procedures | Emergency |  |
|  |  | Elective |  |
| 202 | Types of procedures | Orthopedic |  |
|  |  | Abdominal surgery |  |
|  |  | urologic |  |
|  |  | ENT |  |
|  |  | Ophthalmic |  |
|  |  | Neurosurgery |  |
|  |  | Other ………. |  |
| 203 | Types of anesthesia | GA with ETT |  |
|  |  | GA with facemask |  |
|  |  | GA with LMA |  |
| 204 | Use of caudal block | Yes |  |
|  |  | No |  |
| 205 | Use of IV fluid administration >500ml | Yes |  |
|  |  | No |  |
| 206 | Warmed fluid administered | Yes |  |
|  |  | No |  |
| 207 | Is blood transfused? | yes |  |
|  |  | No |  |
| 208 | Induction agent | Ketamine |  |
|  |  | Propofol |  |
|  |  | Thiopental |  |
|  |  | Ketofol |  |
| 209 | Muscle relaxant | Suxamethonium |  |
|  |  | Vecuronium |  |
|  |  | pancronium |  |
|  |  | Sux and vecuronium |  |
|  |  | Not used |  |
| 210 | Inhalational anesthetics | halothane |  |
|  |  | isoflurane |  |
|  |  | sevoflurane |  |
|  |  | None |  |
| 211 | Analgesic | Morphine |  |
|  |  | Pethidine |  |
|  |  | Fentanyl |  |
|  |  | Other…. |  |
| 212 | Duration of surgery | _______________ |  |
| 213 | Duration of anesthesia | _______________ |  |
